# Fever, Cutaneous Ulcers, and Arthritis Define the MDA5 Phenotype in Indian Patients with Idiopathic Inflammatory Myositis

**DOI:** 10.31138/mjr.33.4.413

**Published:** 2022-12-31

**Authors:** Phanikumar Devarasetti, Irfan Mohammad, Anitha Desai, Liza Rajasekhar

**Affiliations:** Department of Clinical Immunology and Rheumatology, Nizam’s Institute of Medical Sciences, Hyderabad, Telangana, India

**Keywords:** MDA5 dermatomyositis, cutaneous ulcers, RPILD, CADM

## Abstract

**Background:**

In idiopathic inflammatory myositis (IIM), anti-MDA5 (melanoma differentiation-associated gene 5) antibody-associated DM (MDA5 DM) is a distinct subset characterised by presentation with cutaneous involvement, association with ILD and delayed onset of muscle involvement which is believed to be not severe.

**Objectives:**

To study the clinical profile and treatment outcomes of MDA5 DM patients.

**Methods:**

Records of patients fulfilling the ACR/EULAR classification criteria for IIM and testing positive for MDA5 antibody, were retrieved from the ongoingMyoIN registry database of our centre. Clinical, laboratory and treatment data were analysed. Follow-up details were noted. Parameters were compared between survivors and non-survivors using student’s t-test or Mann-Whitney U tests for continuous variables and Chi square test and Fisher’s exact for categorical variables.

**Results:**

Eighteen patients (5 juvenile) were identified. Median age was 30(16–41) years and disease duration at time of diagnosis was 5.5 months with median follow up duration of 18 months (12–31). The prevalence of constitutional symptoms, cutaneous involvement, arthritis, and myositis were 94%, 100%, 78%, and 44% respectively. Ulcers were the most common cutaneous finding. Myositis when present was severe. Interstitial lung disease (ILD) was present in 6 patients which was rapidly progressive (RPILD) in 3. Anti-Ro-52 antibody (n=5) was the most common myositis associated antibody (MAA). There was no difference in the clinical profile of children and adults. Seven patients succumbed. RPILD was a predictor of mortality (p=0.04). Cutaneous relapses were common among survivors but responded well to further therapy.

**Conclusion:**

The MDA5 DM phenotype in this Indian cohort is characterised by fever, cutaneous ulcers and arthritis. RPILD was not uncommon and predicted mortality.

## KEY MESSAGES

1. Skin, joint involvement, and fever is the most common phenotype of MDA5 DM.
2. Cutaneous ulcers are very common. Myositis is not uncommon, and when present, is severe in spite of normal creatine phosphokinase (CPK).
3. RPILD is a predictor of mortality.

## INTRODUCTION

Dermatomyositis (DM) is an idiopathic inflammatory myopathy classically characterised by mild muscle weakness, laboratory or histological evidence of muscle inflammation, skin lesions, and involvement of extra-muscular organs.^1^ DM patients frequently have specific autoantibodies strongly associated with distinct clinical phenotypes, making them useful for prognosis.

Clinically amyopathic dermatomyositis (CADM) is an important subset of DM and includes amyopathic disease (ADM) and hypomyopathic disease (HDM). ADM is defined as the occurrence of hallmark cutaneous findings of DM, without any clinical or laboratory evidence of muscle disease for 6 months or longer.^2^ Population-based epidemiologic studies have suggested that amyopathic DM might account for 20% of the total population of Dermatomyositis (DM) patients.^3^ In 2005, Sato et al described a new autoantibody directed against melanoma differentiation protein-5 (MDA-5) in Japanese CADM patients who presented with rapidly progressive interstitial lung disease but absent muscle weakness.^4^ The information on MDA5 DM from India is scarce.^5,6^ This study informs regarding the clinical profile and outcomes in this disease from a tertiary care University teaching hospital in India.

## MATERIALS AND METHODS

The MyoIN registry is a prospective, multicentre registry from India aimed at studying risk factors for susceptibility, severity and prognosis of inflammatory myopathies.^7^ Registry was initiated in four centres in India to maintain patient follow up data and bio repository of IIM patients. Records of patients with MDA5 positive DM, fulfilling the ACR/EULAR classification criteria for Idiopathic inflammatory myopathies (IIM)^8^ and evaluated at our Institute were retrieved from the MyoIN registry. Demographic and clinical data were noted. Manual Muscle Testing 8 scores (MMT8) were also noted, and severe myositis was defined as functional class III/IV. Muscle biopsy was done in patients who had muscle weakness. Patients with suspected ILD based on symptoms had a baseline high resolution computerised tomography (HRCT) of the chest. Pulmonary function tests (PFT) were done wherever the patient’s condition permitted. Forced expiratory volume in 1^st^ second (FEV1) and forced vital capacity (FVC) was recorded. Baseline 2D Echo is available in all patients. Muscle enzymes (creatine phosphokinase [CPK], lactate dehydrogenase [LDH]), antinuclear antibody (ANA) by indirect immunofluorescence (IIF) were noted. ANA was done using at 1:100 dilution and the pattern was described as homogenous, speckled, nucleolar, cytoplasmic, mixed or others with intensity of ANA above 2+ taken as significant. Myositis specific and associated antibodies (MSA and MAA) were detected using EUROIMMUN EUROLINE kit (Medizinische Labordiagnostika AG) which provides semi-quantitative determination of autoantibodies of the immunoglobulin class IgG to 16 different antigens.^9^ Titres of 2+ and above (semiquantitative values of 25 and above) were taken as positive. Informed consent was obtained from study participants as part of the ongoing MyoIN registry. This study has approval from Nizam’s Institute of Medical Sciences (NIMS) Institutional Ethics Committee (PBAC No 1226/2018).

### Statistics

Categorical variables were described as frequencies and continuous variables as median (interquartile range). Univariate analysis was done using Student’s t-test or Mann Whitney U tests for continuous variables and Chi square test and Fisher’s exact for categorical variables. Comparisons are made as binary groups. Statistical Package of Social Sciences (SPSS) version 21 (IBM SPSS Inc, Chicago, IL) was used, and p value ≤ 0.05 was considered significant.

## RESULTS

Of the 156 IIM patients from our centre in the MyoIN cohort, 18 of 74 DM patients (adult/juvenile) had anti-MDA5 antibodies. Six were less than 16 years at diagnosis (juvenile). **Table 1** summarises the clinical characteristics of these 18 patients.

**Table 1.** Baseline demographic, clinical and laboratory characteristics of MDA5 DM patients.

**Number of patients(n)**	18

**Age(median)(IQR) years**	30(16–41)

**Sex(M/F)**	6/12

**Disease duration at time of diagnosis (median)(IQR)months**	5.5(3–7)

**Skin involvement n (%)**	18 (100)

**Heliotrope rash**	9 (50)
**Gottron’s papules**	8 (44)
**Gottron’s sign**	10 (55)
**Cutaneous ulcers**	11 (61)

**Fever/weight loss**	17 (94)

**Arthritis(%)**	14 (77)

**Symptomatic Interstitial lung disease (%)**	6 (33)
	3 (16)
**RP ILD**	

**Muscle weakness (%)**	8 (44)

**MMT8 in those with myositis (median)(IQR)**	42.5(39.7–45.2)

**Elevated CPK/LDH(n)**	1/4

**ANA (IIF) (%)**	
**Negative**	10 (55)
**Cytoplasmic**	2 (11)
**Speckled**	3 (17)
Others*	3 917)

**MDA5 intensity(median)(IQR)**	77.5(46–88)

**Dual MSA positivity(n) (anti-SRP/anti-PL7)**	2 (1/1)

**Anti-Ro-52 positivity (%)**	5 (28)

**Duration of follow up (median) (IQR)months**	18(12–31)

IQR: Inter quartile range;

*: Mixed (N+C)(Centromere/speckled/Homogenous)

**Figure 1.**
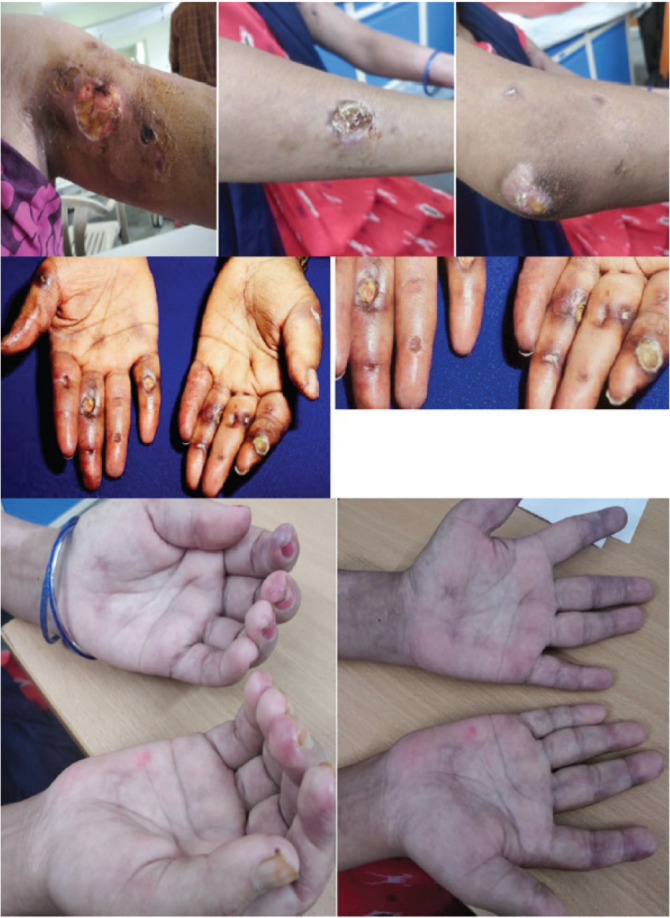
Skin manifestations in MDA5 DM. Top row: Punched out ulcers on arm, forearm, and extensor aspect of elbow. Middle row: Extensive ulcers in PIP AND DIP creases. Lower row: Vasculitic rash over fingers with left index finger digital ischemia and erythematous palmar papules along the ulnar side.

All patients had cutaneous manifestations, which were invariably present at onset. Cutaneous ulcers were the commonest finding, seen in 11 patients. These ulcers were deep and seen predominantly on upper limbs over metacarpophalangeal joints and elbows (**Figure 1**). Gottron’s sign, heliotrope rash, and Gottron’s papules were the next most frequent manifestations. Inverse Gottron’s and palmar papules were seen in 3 and 4 patients respectively. Panniculitis and the mechanic’s hand were present in 3 patients each. Digital pits, digital ischemia, vasculitic rashes and panniculitis were seen more commonly in adults than in juveniles. Cutaneous manifestations, other domain involvement, and treatment details with outcomes are summarised in **Table 2**. Systemic manifestations of fever and weight loss were universal.

**Table 2.** Clinical profile and details of cutaneous manifestation.

**No**	**Age Sex**	**Time to diagnosis (months)**	**Skin**	**Myositis**	**ILD**	**Arthritis**	**Treatment**	**Outcome**
1	25/M	7	GP, GS, DP, alopecia, CU, panniculitis	Y	N	Y	GC, MTX	Remission
2	16/F	7	HR, GP, alopecia, CU	N	N	Y	GC, MTX	Remission
3	45/F	7	Palmar papule, inverse Gottron’s sign, RP, DP, VR, CU	N	N	Y	GC, MTX, AZA	Remission
4	27/M	3	HR, GP, alopecia	Y	N	Y	GC, MTX	Remission
5	55/F	2	HR, palmar papule, SS, MH	N	Y	Y	GC, MTX	Remission
6	8/F	4	GS, alopecia	N	N	Y	GC, MTX, CYC	Remission
7	28/F	6	HR, GS, MR, panniculitis, Calcinosis	Y	N	N	GC, AZA, MMF, MTX, IVIG, RTX	Remission
8	35/F	5	HR, GS, Calcinosis, Generalized hyperpigmentation	Y	N	N	GC, MTX	Remission
9	16/M	6	GS, CU	N	N	Y	GC, MTX	Remission
10	23/F	3	HR, GP, MR, palmar papules, inverse Gottron, DU, panniculitis cuticular infarct, VR, CU	Y	N	Y	GC, MTX, MMF, CYC, RTX	Remission
11	35/F	1	HR, VR, MR, alopecia	N	Y	Y	GC, MTX, AZA	Remission
12	32/M	14	Generalised hyperpigmented rash	Y	N	N	GC, MTX	Death (Rhabdomyolysis, Sepsis, lobar pneumonia
13	38/F	3	GP, GS, MR, DU, MH, CU, digital gangrene	Y	Y	Y	GC, MTX, CYC	Death(MRSA Sepsis )
14	3/F	6	GP, GS, palmar papules, CU	N	Y	Y	-	Death (RPILD)
15	40/F	120	GS, alopecia, CU	Y	N	Y	GC, MTX	Death (Active disease, Pneumonia (Klebsiella)
16	10/F	18	HR, GP, GS, MR, alopecia, poikiloderma, oral ulcer	N	N	Y	GC, MTX	Death (Sepsis / pneumonia)
17	50/M	4	HR, MR, SS, poikiloderma, CU	N	Y	Y	GC, PLEX, CYC, TAC, RTX	Death (RPILD) Pneumomediastinum COVID infection)
18	48/M	2	GP, GS, VR, MH, CU	N	Y	N	GC, MTX, MMF	Death(RPILD)

GP: Gottron’s papule; GS: Gottron’s sign; HR: heliotrope rash; MR: malar rash; MH: Mechanic’s hands; CU: cutaneous ulcers; SS: shawl sign; VR: vasculitic rash; RP: Raynaud’s phenomenon; DP: digital pits; DU: digital ulcer; GC: Glucocorticoids; MTX: Methotrexate; AZA: Azathioprine; MMF: Mycophenolate; CYC: Cyclophosphamide; RTX: Rituximab; TAC: Tacrolimus; PLEX: Plasmapheresis; IVIG: intravenous immunoglobulin

Arthritis was seen in 14 patients, 6 of whom had simultaneous joint and skin manifestations. The arthritis was symmetrical, polyarticular and non-deforming.

Severe muscle weakness was noted in all the 8 patients with myositis. Onset of myositis preceded skin involvement in 3. Median time to onset of myositis was 4 months. Elevation of muscle enzymes beyond the upper limit of laboratory normal was noted for LDH (median 403(230–590) IU/ml) but not for CPK (median 79.5(45–143) IU/ml). In the 8 patients in whom muscle biopsy was available perifascicular atrophy was seen in 6, fibre degeneration and regeneration in 5 and fibre necrosis in 2.

Interstitial lung disease was noted in 6 patients. Nonspecific interstitial pneumonia (NSIP) was the predominant pattern (n=4). The ILD was rapidly progressing (RP ILD) and fatal in three, including a 3-year-old child. Pneumomediastinum was seen in one. Time to death was between one and three months. One patient did not respond to multimodality therapy with high dose steroids, Rituximab, Plasmapheresis, Tacrolimus, Cyclophosphamide and finally succumbed with preterminal COVID 19 infection.

Clinically amyopathic dermatomyositis (CADM) as defined by absence of clinical evidence of myositis was seen in 10 patients. Seven patients were hypo myopathic (HDM) (named as such due to presence of elevated muscle enzymes, in this case only LDH not CPK) and 3 were amyopathic (neither clinical nor biochemical evidence of myositis). None of these 10 patients developed clinical muscle weakness in the follow up period of 18 months. Arthritis either simultaneously or after onset of skin involvement was seen in all these CADM patients. All three RPILD patients belonged to the CADM group. 2D echo was normal in all except in one patient, who had mild pulmonary artery hypertension.

ANA by IIF was not detected in most of these patients, anti-Ro-52 antibodies were the only (n=5) MAA seen. The median MDA5 intensity online blot was high (77.5[46–88]), but not different between patients who were treatment naive and treatment experienced.

No significant differences were found in clinical, laboratory profile and outcomes between juvenile and adult MDA5 patients. Clinical and laboratory profile of survivors and non-survivors summarised in **Table 3**. RPILD (p=0.04) was significantly more frequent in non-survivors and the commonest cause of death. Sepsis was the next most frequent cause of death.

**Table 3.** Comparison of survivors and non-survivors.

	**Survivors**	**Non-survivors**	**p value**

No. Of patients	11	7	

Age(median)(IQR) yrs	27(19–35)	38(21–44)	0.53

Females (%)	8 (72)	4 (57)	

Disease duration (median)(IQR)mo	5(3–6.5)	6 (3.5–16)	0.37

Fever/weight loss (%)	10 (90)	7 (100)	0.61

Skin involvement (%)	11 (100)	7 (100)	

Heliotrope rash (%)	7 (63)	2 (28)	0.33
Gottron’s papules (%)	4 (36)	4 (57)	0.63
Gottron’s sign (%)	5 (45)	5 (71)	0.37
Cutaneous ulcers (%)	6 (54)	5 (71)	0.63
Digital pits (%)	3 (27)	1 (14)	0.62
Digital ischemia (%)	1 (9)	2 (28)	0.52
Vasculitic rash (%)	3 (27)	1 (14)	0.62

Arthritis (%)	9 (81)	5 (71)	0.51

ILD (%)	2 (18)	4 (57)	0.08

RP ILD (%)	0	3 (42)	0.04*

Muscle involvement (%)	5 (45)	3 (42)	0.67

MMT8(median)	65(45–80)	58(42–80)	0.67
CPK	41(34–80)	91(42–97)	0.42
LDH	403(279–571)	406(235–514)	0.86

ANA positivity (%)	5 (45)	3 (42)	0.64

MDA5 intensity (mean/SD)	71(43–99)	73(56–90)	0.72

Anti-Ro-52 positivity (%)	3 (27)	2 (28)	0.67

All patients received glucocorticoids and methotrexate for arthritis and myositis. Switch of immunosuppression to either mycophenolate mofetil / cyclophosphamide/rituximab was done for refractory cutaneous disease in two survivors.

Events during follow-up included development of calcinosis in 3 patients, tuberculosis in 2 (cutaneous in 1 and disseminated in 1) and stroke in one juvenile patient (demyelinating illness responded to high dose steroids and CYC pulse therapy). At median follow up of 18 months, all survivors had clinical remission in skin, joint and muscle domains.

## DISCUSSION

We report here our experience with anti-MDA5 antibody associated subset of inflammatory myositis. As expected, most of the patients presented with predominant skin involvement. The cutaneous manifestations varied from classic DM rashes and cutaneous ulcers in most of the patients to some uncommon manifestations such as calcinosis, panniculitis, and digital ischemia. Fever and weight loss were almost universal. Symmetrical non-deforming polyarthritis was commonly seen. Myositis was not uncommon, seen early in disease and severe with normal CPK in most of the patients. The combination of skin and joint involvement was most frequent in our group and responded well to therapy. Rapidly progressive ILD was a predictor of mortality.

In previous Indian studies which mention MDA5 DM in their descriptions of IIM patients a prevalence varying from almost 9% in the multicentre MyoIN cohort,^10^ 17% from North India^5^ to 24% in the current cohort which is a subset of the MyoIN including patients from only our centre. Reports from other centres in Japan, Europe and North America are similar.^11–13^

The phenotype of CADM defined initially as cutaneous manifestations of DM in absence of myositis^2^ subsequently came to be associated with RPILD and presence of anti-MDA5 in serum of these patients.^3^ However in our cohort of anti-MDA5 DM we report a prevalence of 44% for myositis, very early after the first symptom of the disease which is almost similar to the reported prevalence of 56% from another Indian cohort of MDA5 DM.^6^

In our cohort, cutaneous ulcers were the most common skin manifestation and were seen in 61% consistent with the prevalence of 40–80% reported in literature.^6,11,14,15,16^ These ulcers were seen on typical locations over knuckles and elbows as described in various MDA5 DM cohorts. However, the typically reported classical DM rashes such as Gottron’s papules, heliotrope rash, Gottron’s sign were seen in only 50% of our cohort compared to 68% reported by Dunga et al.^6^ We also report the presence of digital pits, digital gangrene, and vasculitic rashes. These do not find a mention in previous reports from India.^5,6,10^ The largest report of anti-MDA5 positive patients till date referred to 121 patients from France reported three phenotypic clusters: one with RPILD and poor prognosis(18%), another with skin and rheumatic manifestations and a good prognosis (55%) and a third with severe myositis and cutaneous vasculopathy(26%).^15^ We had two clusters, skin and rheumatic manifestation being commoner (77%) compared to the RPILD phenotype (16%).Digital necrosis has been used to define one of three clusters. Digital necrosis also finds mention in another report on MDA5 DM.^17^

We also make note of the presence of calcinosis and panniculitis. Calcinosis has been reported previously^15,18,19^ and is probably more common in males and those with severe skin involvement.^15^ MDA5 positivity was predictor of calcinosis in JDM cohort.^2^ Panniculitis as reported in literature^19,20^ was seen in a few patients of our cohort. Fever and weight loss was reported to have an association with MDA5 DM.^4,6^ Fever as a disease manifestation was very common (94%) in our cohort too. However, in studies from other parts of the world prevalence was lower and ranged between 30–40%.^11,15,19,20^ Oral ulcers and eyelid oedema reported in MDA5 DM ^5^ were not seen in any of our patients.

The frequency of myositis was consistent with that in published literature.^15,19^The myositis was however severe in all and not associated with the severe vasculopathy reported by Allenbach.^15^ Elevation of CPK was very infrequent compared to higher values (36% to 41%) reported by others.^15,19^

We report frequent arthritis (77%) which was polyarticular, symmetrical, and non-deforming. A prevalence ranging from 50 to 80% has been reported in Indian, East Asian, and western cohorts.^4,6,15,19^

ILD was present in one third of the patients. A quarter had RPILD which was a predictor of mortality. No patient with RPILD had myositis, hence they could be called CADM. Dunga et al. from north India reported ILD in half of patients with MDA5 DM, one third had RPILD.^6^ In a Japanese cohort a prevalence of RPILD of 57% was reported among 28 MDA5 DM.^20^ Spontaneous pneumomediastinum is described with RPILD^21^ and considered a poor prognostic factor. One patient in our cohort with RPILD has spontaneous pneumomediastinum. Prevalence of MDA5 DM in JDM in Indian patients ranges from 28% (5 of 18) in our cohort to 10–14% (5 of 25) JDM from the previous study.^5,6^ From UK a prevalence of 7.4% among 285 JDM has been reported.^23^As in other cohorts skin ulcers and arthritis were common.^24–28^ Association of polyarthritis with MDA5 JDM has been reported in a German cohort of 196 patients with JDM.^27^ The North American registry of JDM also reported weight loss and arthritis in MDA5 JDM.^28^ The reported prevalence of RPILD varies from 19% in the UK JDM^23^ to 30% in the East Asian cohort.^29^ The one case with RPILD in our cohort of MDA5 JDM is probably the youngest described till date. In another Indian study among MDA5 JDM, none had ILD.^6^

While MSA are considered to be mutually exclusive, we found high intensity (>25) SRP and PL7 in one patient each. Four other patients had a lower-intensity additional MSA. Double positivity has been reported in three case reports from Japan and the USA. Two had associated PL7 and one EJ antibody.^30,31^ All these three cases had RPILD. Our case with PL7 overlap has minimal ILD, while the patient with previously unreported association with SRP had severe myositis with rhabdomyolysis, but no ILD.

We did not find any association of anti-Ro52 antibodies with RPILD and cutaneous involvement as mentioned in previous Indian and Asian studies.^6,32,33^

Mortality is high and early in RPILD, similar to various published cohorts from India and the rest of the world.^6,13,15,17,21,34^ Infections causing death have been reported both in our and previously published study from North India.^34^

## CONCLUSION

MDA5 DM in Indian IIM patients presents with cutaneous manifestations which includes ulcerations on or around joints and the classic rash of Dermatomyositis. Calcinosis in Indian MDA5 DM is being reported for the first time. Fever and weight loss is common. Myositis early in the disease is not infrequent and is severe. ILD, including RPILD, is also noted in half the patients. Mortality is high and predicted by RPILD.

## Data Availability

The datasets generated during and analysed during the current study are available from the corresponding author on reasonable request.
